# Using secure artificial intelligence agents integrated within the electronic medical record for the evaluation of blood culture appropriateness—Northern California, 2025

**DOI:** 10.1017/ice.2025.10349

**Published:** 2026-02

**Authors:** Guillermo Rodriguez-Nava, Timothy Keyes, Nerissa Ambers, Eugenia Miranti, Erika Paola Viana-Cardenas, Wajeeha Tariq, Mindy Marie Sampson, Jorge Luis Salinas

**Affiliations:** 1 Division of Infectious Diseases & Geographic Medicine, Stanford University School of Medicine, Stanford, CA, USA; 2 Department of Medicine, Stanford University School of Medicine, Stanford, CA, USA; 3 Department of Nursing Information & Informatics, Stanford Health Care, Stanford, CA, USA

## Abstract

We evaluated large language model (LLM)-based agents integrated with the electronic medical record to assess blood culture appropriateness. While sensitivity was high, specificity remained low. Performance was shaped by prompt phrasing, sycophantic behavior, and semantic triggers, reflecting both the potential and limitations of LLMs in real-world clinical decision support.

## Background

Barriers to effective antimicrobial stewardship and infection prevention include the lack of structural frameworks, guidelines, training, funding, and access to manpower, diagnostics, and information technology. Transformer-based large language models (LLMs) represent a major advance in natural language processing, with potential to augment clinical workflows and address workforce gaps in infectious diseases.

Prospective audit and feedback is a widely used stewardship strategy but remains labor-intensive, especially in resource-constrained settings.^1,2^ LLMs have shown human-like clinical reasoning in exams and simulated cases and may support clinical decision-making.^3^ However, due to privacy and data access constraints, few studies have evaluated their use in real-world healthcare settings.^4^ We aimed to evaluate the accuracy of LLM-based conversational agents in auditing blood culture appropriateness using real patient records.

## Methods

As part of our Blood Culture Stewardship program, which includes random appropriateness review and provider feedback, an infectious diseases provider (G.R.N.) reviewed 105 blood culture orders (May–December 2024). The reviews were guided by a published algorithm adapted to our institution (Table 1).^5^ These adjudications were then used for this study. Orders labeled “maybe appropriate” were excluded, as were those placed within 48 hours of a prior culture, which were predefined as inappropriate. After these exclusions, 67 orders remained for LLM evaluation (31 appropriate, 36 inappropriate).

**Table 1.** Institutional guidance for appropriate blood culture ordering

Indications for INITIAL blood cultures (BCx) in non-neutropenic adult patients		
BCx not indicated	BCx may be indicated	BCx indicated
Syndromes with low risk of bacteremia and severe sepsis criteria not met Cellulitis Colitis (including *C. difficile*) Cystitis Aspiration pneumonitis CAP, aspiration PNA Diabetic foot infection Non-vertebral osteomyelitis Uncomplicated cholecystitis Uncomplicated diverticulitis Uncomplicated pancreatitis Viral illness Isolated post-operative fever within 48 hours Surveillance blood cultures for any reason (e.g., patient with central line needing TPN) Fever or leukocytosis explained by a non- infectious cause (e.g., drug withdrawal) Non-infectious syndromes COPD exacerbation Heart failure	New or persistent isolated: tachycardia OR leukocytosis/leukopenia OR hypotension OR fever Assess for infectious and non-infectious causes that can explain findings before ordering BCx Obtain cultures from non-blood sites as indicated first	Severe sepsis (suspected infection AND SIRS AND associated organ dysfunction) Systemic signs of infection AND high risk for endovascular infection (e.g. prosthetic valve, ICD/pacemaker, vascular graft) or with central venous catheter (short or long term) Systemic signs of infection AND asplenia Suspected or confirmed infection with the following syndromes that are associated with concomitant bacteremia: Catheter-related bloodstream infection Cholangitis Endocarditis/endovascular infection (e.g. septic thrombophlebitis) Meningitis Non-traumatic septic arthritis Pyelonephritis if urine not available Severe pneumonia Vertebral OM/discitis, epidural abscess
	**BCx likely not indicated**	
	• Persistent fever or leukocytosis AND 2 sets of negative BCx within 24–48 hours in patients without localizing signs of infection or no clear source of infection	
		**Draw 2 sets of peripheral BCx**
**The algorithm is not a substitute for clinical judgment. If you have any questions regarding this algorithm, please contact the Infection Prevention Department.**		

This guidance was developed as part of the Johns Hopkins Prevention Epicenter Blood Culture Stewardship Collaborative. The content of the algorithm has been adapted from Fabre V, Sharara SL, Salinas AB, Carroll KC, Desai S, Cosgrove SE. Does This Patient Need Blood Cultures? A Scoping Review of Indications for Blood Cultures in Adult Nonneutropenic Inpatients. Clin Infect Dis. 2020, PMID: 31942949.

BCX, blood cultures; COPD, chronic obstructive pulmonary disease; ICD, implantable cardioverter-defibrillator; OM, osteomyelitis; SIRS, systemic inflammatory response syndrome; TPN, total parenteral nutrition.

**Figure 1.**
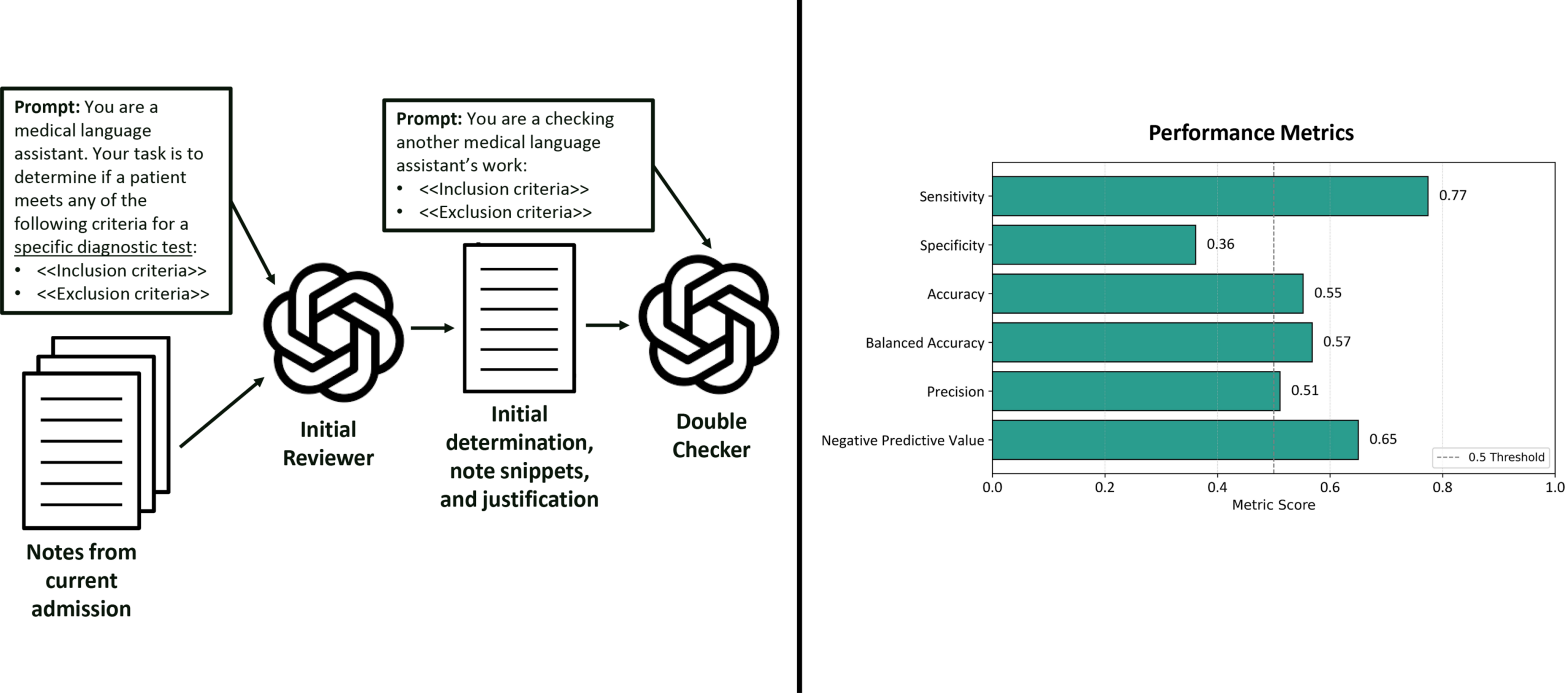
Workflow and performance metrics of secure AI agents for blood culture appropriateness assessment. Notes from the current admission were reviewed by an initial reviewer agent prompted to assess for specific inclusion/exclusion criteria. A double-checker agent then independently reassessed the output. Right: performance metrics compared to expert adjudication.

Stanford University has collaborated with industry partners to deploy secure LLM-based conversational applications with direct access to electronic medical records for medical applications. Using these tools, we developed two task-specific artificial intelligence (AI) agents to assess blood culture appropriateness using our institutional algorithm. The *Initial Reviewer* agent (gpt-4o-mini; OpenAI) scanned the notes for mentions of predefined appropriateness or inappropriateness criteria. A second, more powerful agent, the *Double-Checker* (gpt-o1-mini; OpenAI), then reviewed and, if necessary, corrected the initial classification (Figure 1). All History & Physical and progress notes from the current admission were individually reviewed by the AI agents, from admission to the blood culture order. If any note for a given blood culture order was flagged as meeting appropriateness criteria, the entire order was classified as *appropriate*; otherwise, it was labeled *not appropriate*. Each classification included explanations with note excerpts and applied criteria, allowing review of agent reasoning. Performance metrics were calculated using infectious disease expert adjudication as the gold standard, with expert-defined appropriate orders considered true positives and inappropriate orders true negatives.

## Results

The number of notes per patient ranged from one to nearly 500. The *Initial Reviewer* agent consistently agreed with care team decisions when made aware that it was evaluating the appropriateness of blood culture orders. Conversely, the *Double-Checker* agent overrode the *Initial Reviewer* in nearly all cases when explicitly informed that the *Initial Reviewer* might be prone to error.

To address this limitation, we reframed the prompts: rather than asking the *Initial Reviewer* to assess the appropriateness of a blood culture order directly, we asked whether the patient met predefined criteria (from the algorithm) for a specific diagnostic test. The *Double-Checker* was then instructed to review the *Initial Reviewer*’s output using the same criteria, without assumptions or reference to the specific diagnostic test being evaluated. The prompts used can be found in Supplementary Material 2. With this approach, sensitivity was 77.4%, indicating the agents identified most appropriate orders. Specificity was low at 36.1%, reflecting limited ability to exclude inappropriate orders. Balanced accuracy was 56.8%, suggesting limited discrimination. Additional metrics included 55.2% accuracy, 51.1% precision (positive predictive value), and 65.0% negative predictive value (Figure 1). We also tested a consensus-based approach, in which orders were classified as appropriate only if a defined proportion of notes were deemed appropriate by the AI agents. A majority threshold (>50%) increased specificity to 97% but reduced sensitivity to 0%; a 10% threshold yielded 50% specificity and 71% sensitivity.

After reviewing the AI agents’ justifications, they continued to frequently over-classify orders as appropriate by aligning with the care team. The Supplementary Table presents a sample of cases with AI classifications, expert adjudication, and associated documentation. Semantically influential terms more frequently appeared in agent justifications. For instance, “sepsis” was mentioned at least once in 34% of appropriate classifications, compared to 9% of not appropriate.

## Discussion

In this pilot study, AI agents demonstrated a clear trade-off between sensitivity and specificity when assessing blood culture appropriateness. Their determinations were shaped by prompt framing, with frequent agreement when asked to assess appropriateness directly or to review another agent labeled as error-prone. Even with neutral prompting, the agents tended to align with care team decisions, and their classifications were further influenced by semantically charged terms, particularly “sepsis.”

The agents identified most appropriate orders, consistent with our conservative approach. Consensus-based strategies improved specificity but sharply reduced sensitivity. In contrast, our prior work on central line-associated bloodstream infections (CLABSI) showed that manually adding missing clinical information improved both sensitivity and specificity.^3^ Blood culture appropriateness was more affected by contextual interpretation and by the thresholds used to classify an entire order based on note-level determinations. In the CLABSI task, performance rose from 80% sensitivity and 35% specificity with limited input to 90% and 75% once additional information was provided. The improvement with human input illustrates how expert oversight can guide models toward greater accuracy, supporting their role as screening tools with human-in-the-loop review.

LLM-based conversational agents have demonstrated remarkable capabilities across a wide range of natural language understanding tasks and processing in medicine.^4^ These systems are typically trained through supervised fine-tuning followed by reinforcement learning guided by human preferences to improve performance and alignment. During this process, crowd-workers select their preferred response from multiple options, but these labels are not always reliable. Because human feedback guides the training process, these systems may exhibit a phenomenon named *sycophancy*.^6^ This behavior may present in different forms, such as giving inaccurate responses to match user expectations, offering inappropriate recommendations when prompted, or accepting incorrect assumptions without correction.^7^ In our study, agents almost always agreed with care teams when instructed to directly evaluate blood culture appropriateness. Similarly, the *Double-Checker* frequently disagreed when told it was reviewing the *Initial Reviewer’s* work. While LLM developers work to reduce sycophancy, users can mitigate it through prompt engineering, such as using neutral phrasing, authorizing disagreement, or instructing the model to take a contrarian role.

Semantic triggers also influenced the agents’ decisions. These models are trained to predict the next word in a sequence, enabling them to generate coherent, contextually appropriate responses. As a result, they often associate specific terms with expected clinical actions, even when those associations are not justified.^8^ In this study, documentation of “sepsis” was a prominent trigger. Blood cultures have a moderate yield in severe sepsis and are a required metric under the Centers for Medicare and Medicaid Services’ SEP-1 bundle.^5^ However, sepsis is difficult to diagnose given its nonspecific presentation. Sepsis is misdiagnosed in 25% of acute heart failure cases, and nearly half of presumed ICU sepsis lacks confirmed infection.^9,10^ The AI agents likely associated mentions of “sepsis” with blood culture use, regardless of whether diagnostic criteria were met.

Our study has limitations. It was conducted at a single center with a modest sample size, limiting generalizability and power. Model performance was also influenced by prompt design, highlighting the role of prompt engineering in applied settings. Lastly, our initial classification strategy of labeling an order appropriate if any note met criteria likely contributed to the initial overclassification and low specificity. In tasks with sharp trade-offs such as blood culture appropriateness, classification thresholds may need to be adjusted to balance sensitivity and specificity appropriately.

In conclusion, LLM-based review of blood culture appropriateness demonstrated a different trade-off between sensitivity and specificity compared to other infectious diseases tasks. Prompt framing, sycophancy, and semantic cues were key influences. Real-world applications may require task-specific adjustments to balance sensitivity and specificity according to clinical needs, with human-in-the-loop oversight remaining essential.

## Supporting information

Rodriguez-Nava et al. supplementary material 1 Rodriguez-Nava et al. supplementary material

Rodriguez-Nava et al. supplementary material 2 Rodriguez-Nava et al. supplementary material

## Supplementary material

The supplementary material for this article can be found at https://doi.org/10.1017/ice.2025.10349
