# Supplementary material for: Using secure artificial intelligence agents integrated within the electronic medical record for the evaluation of blood culture appropriateness—Northern California, 2025
Source: Infect Control Hosp Epidemiol. 2025 Nov 11;47(2):206–9. doi: 10.1017/ice.2025.10349 (PMC12926330; doi:10.1017/ice.2025.10349)
Supplement: Rodriguez-Nava et al. supplementary material 1 — Rodriguez-Nava et al. supplementary material [file S0899823X25103498sup001.pdf]

INITIAL REVIEWER PROMPT

This patient is being evaluated for the appropriateness of ordering a specific medical test.

We need your help reviewing the following **inclusion** and **exclusion** criteria to determine whether the test is **appropriate** or **inappropriate** for this patient.

Patient Clinic Note Review

Below is a recent **clinic note** describing the patient's presentation prior to the test order. Carefully **review the note** and **check each inclusion and exclusion criterion** before making a determination.

<<Insert Note>>

Inclusion Criteria

The test should be ordered if the patient meets **any** of the following criteria:

1. Severe Sepsis

Defined as **suspected infection** AND **associated organ dysfunction** AND **meeting SIRS criteria**.
The **SIRS criteria** include at least two of the following:
- *Temperature:* <36°C (96.8°F) or >38°C (100.4°F)
- *Heart Rate:* >90 bpm
- *Respiratory Rate:* >20 breaths/min or $PaCO_2$ <32 mmHg
- *White Blood Cell Count:* <4,000 or >12,000 cells/mm^3, or >10% immature forms or bands

2. Systemic Signs of Infection + High Risk of Endovascular Infection

Includes any of the following risk factors:
- ICD/Pacemaker
- Vascular graft
- Prosthetic heart valves or valve repair materials
- History of infective endocarditis
- Valvulopathy
- Endovascular thrombi
- Presence of a central venous catheter

3. Systemic Signs of Infection + Asplenia

4. Suspected or Confirmed Infection with Specific Syndromes

The following conditions are strongly associated with **concomitant bacteremia** and warrant the test:
- **Catheter-related bloodstream infection**
- **Cholangitis**
- **Endocarditis / Endovascular infection** (e.g., septic thrombophlebitis)
- **Meningitis**
- **Non-traumatic septic arthritis**
- **Pyelonephritis (only if urine sample unavailable)**
- **Severe pneumonia** (ONLY if requiring ventilation or present in a patient with septic shock/severe sepsis)
- **Vertebral osteomyelitis (OM), discitis, or epidural abscess**

Exclusion Criteria

The test is **not** recommended if the patient meets any of the following conditions:

5. Syndromes with Low Risk of Bacteremia

Unless severe sepsis is suspected, the test is **not indicated** for these conditions:

- **Cellulitis**
- **Colitis (including C. difficile)**
- **Cystitis**
- **Aspiration pneumonitis**
- **Community-acquired pneumonia (CAP)**
- **Aspiration pneumonia**
- **Diabetic foot infection**
- **Non-vertebral osteomyelitis**
- **Uncomplicated cholecystitis**
- **Uncomplicated diverticulitis**
- **Uncomplicated pancreatitis**
- **Viral illness**

- **Isolated post-operative fever within 48 hours**

6. “Surveillance” testing

Routine or precautionary orders for the test are **not appropriate** (e.g., patient with central line needing TPN).

7. Fever or Leukocytosis Due to Non-Infectious Causes

If fever or leukocytosis is **attributable to a non-infectious cause** (e.g., drug withdrawal), the test is **not indicated**.

8. Non-Infectious Syndromes

The test is **not indicated** in cases of:
- **COPD exacerbation**
- **Heart failure**

Final Request

Using the above criteria, review the patient’s note and **answer the following questions**.

Eligibility criteria

- Does the patient reach criterion 1? If so, provide the date of the event that supports your answer and a quote from the note to support.
- Does the patient meet criterion 2? If so, provide the date of the event that supports your answer and a quote from the note to support.
- Does the patient meet criterion 3? If so, provide the date of the event that supports your answer and a quote from the note to support.
- Does the patient meet criterion 4? If so, provide the date of the event that supports your answer and a quote from the note to support.

Exclusion criteria

- Does the patient reach criterion 4? If so, provide the date of the event that supports your answer and a quote from the note to support.
- Does the patient meet criterion 5? If so, provide the date of the event that supports your answer and a quote from the note to support.

- Does the patient meet criterion 6? If so, provide the date of the event that supports your answer and a quote from the note to support.
- Does the patient meet criterion 7? If so, provide the date of the event that supports your answer and a quote from the note to support.

Ultimate determination

Review the answers to the preceding questions. Based on their response, provide output for the following question:

For this patient, is the test order appropriate or inappropriate?
If appropriate, respond with True. If not, respond with False.

Additionally, provide a **justification** for your response. The justification **must include**:
1. **A direct quote** from the patient's clinic note (preferably the full paragraph with the supporting evidence).
2. **The exact date (month, day, year)** on which the quoted criteria were noted.
3. The index of the criteria (1-8) supporting the answer.

Do not infer or interpret beyond the provided criteria. Strictly follow the inclusion/exclusion rules without assumptions. Note that many of the orders for this test are placed inappropriately - so it is okay to disagree with a patient's care team if their note does not adequately document the required criteria.

DOUBLE CHECKER PROMPT

You are tasked with **verifying the accuracy** of another assistant's evaluation of whether a test order is appropriate.

The assistant is **sometimes correct** and **sometimes incorrect**—you **must check its reasoning carefully** without assuming it is either right or wrong by default.

Assistant's Justification:

The assistant provided the following justification:

<<INSERT_JUSTIFICATION_HERE>>

Your job is to **determine whether the justification is correct** based on the explicit criteria for requiring a blood culture.

Explicit Criteria for Ordering the test

The test is **only appropriate** if at least **one** of the following conditions is **explicitly present** in the patient's chart:

1. Severe Sepsis

Defined as:
- **Suspected infection** AND
- **SIRS criteria** (at least two of the following):
- *Temperature:* <36°C (96.8°F) or >38°C (100.4°F)
- *Heart Rate:* >90 bpm
- *Respiratory Rate:* >20 breaths/min or $PaCO_2$ <32 mmHg
- *White Blood Cell Count:* <4,000 or >12,000 cells/mm^3, or >10% immature bands
- **Associated organ dysfunction**

2. Systemic Signs of Infection + High Risk of Endovascular Infection

- **Presence of a central venous catheter** (short or long term)
- **OR** one of the following conditions:
    - Prosthetic heart valve

  - ICD/Pacemaker
  - Vascular graft
  - History of infective endocarditis
  - Valvulopathy
  - Endovascular thrombi

3. Systemic Signs of Infection + Asplenia

4. Suspected or Confirmed Infection with a High Risk of Concomitant Bacteremia

- Catheter-related bloodstream infection
- Cholangitis
- Endocarditis / Endovascular infection (e.g., septic thrombophlebitis)
- Meningitis
- Non-traumatic septic arthritis
- Pyelonephritis (only if **urine sample is unavailable**)
- **Severe pneumonia** (ONLY if requiring ventilation or occurring in septic shock/severe sepsis)
- Vertebral osteomyelitis (OM) / Discitis / Epidural abscess

Verification Task

1. **Does the assistant's justification explicitly mention one of the above criteria?**
   - **If YES**, verify that the assistant correctly cited the information from the chart.
   - **If NO**, the response is incorrect—flag any **assumptions, implications, or missing explicit evidence**.
2. **Was the assistant's reasoning correct and based entirely on the given criteria?**
   - If the assistant inferred or assumed a condition without direct support, the response is incorrect.
3. **Final Answer:**
   - Respond with **True** if the patient **meets at least one explicit criterion** for the test.

- Respond with **False** if none of the criteria are met.

4. **Provide a Justification:**
   - If **True**, cite the **exact sentence** from the justification that justifies the test order.
   - If **False**, explain **why the assistant was incorrect**, identifying any missing or misinterpreted criteria.

Important:

- **Strictly follow the inclusion criteria.** Do not make inferences beyond what is explicitly stated.
- **If the assistant made an assumption, you must explicitly point it out as incorrect.**
- **Your justification must reference the exact wording from the patient's chart whenever possible.**
