# Supplementary material for: Using secure artificial intelligence agents integrated within the electronic medical record for the evaluation of blood culture appropriateness—Northern California, 2025
Source: Infect Control Hosp Epidemiol. 2025 Nov 11;47(2):206–9. doi: 10.1017/ice.2025.10349 (PMC12926330; doi:10.1017/ice.2025.10349)
Supplement: Rodriguez-Nava et al. supplementary material 2 — Rodriguez-Nava et al. supplementary material [file S0899823X25103498sup002.pdf]

Supplementary Table. Sample of Blood Culture Order Classifications by AI Agents Compared to Expert Adjudication, Including Note Type and Testing Indication

Culture Result	Testing Indication	Expert Adjudication	Initial Reviewer Classification	Double-Checker Classification	Note type+	Initial Reviewer Justification
**Negative**	Follow-up cultures for Enterococcus faecalis bacteremia after 72 hours	Appropriate	Not appropriate	Not appropriate	Progress Notes	The test order is not appropriate based on the provided clinic note and criteria. The patient does not meet any inclusion criteria indicative of a severe infection or high risk of bacteremia. Specifically:<br><br>1. There is no evidence the patient has severe sepsis (criterion 1). No SIRS criteria are mentioned; blood cultures were negative for infection ("7/14 BCx: NGTD").<br>2. The patient has no documented systemic signs of infection or criteria from criterion 2 that would suggest a high risk for endovascular infection (e.g., no ICD/pacemaker, vascular graft, or prosthetic valves).<br>3. No documentation supports criterion 3; there's no indication of asplenia.<br>4. There are no indicators of any infection syndromes from criterion 4.<br><br>Furthermore, exclusion criteria are also met (specifically criterion 5), as the patient does not present with severe sepsis, nor any of the high-risk infection syndromes, limiting them from undergoing this specific test.
**Positive**	Follow-up cultures for Staphylococcus aureus bacteremia after 72 hours	Appropriate	Not appropriate	Not appropriate	Progress Notes	The test order is inappropriate because the patient does not meet any inclusion criteria for testing and faces exclusion criteria. This patient is evaluated for acute low back pain and exhibits increased pain and limited mobility during daily activities, which suggests issues regarding physical function rather than systemic infection. Furthermore, the patient has documented low back pain and a pain score that increased but does not satisfy the severe infection or sepsis criteria. The note indicates conditions such as "Patient reported increased pain in back, especially LLE with increased time in standing," which fits exclusion criteria for syndromes with low risk of bacteremia (criterion 5), as well as lack of evidence for systemic infection. Hence, the test is classified as inappropriate.

**Negative**	Follow-up cultures after blood cultures with GPCs in a patient with a central line	Appropriate	Appropriate	Not appropriate	Progress Notes	The patient meets criteria from the inclusion list. For criterion 4, the patient shows signs of a suspected or confirmed infection with a specific syndrome, specifically a **catheter-related bloodstream infection** given the positive MRSA screen and blood cultures. The relevant quote is: "11/14 Blood cx: Candida Krusei 11/14 MRSA Screen: Positive 11/15 MRSA PCR: Positive 11/16 BAL cx: MRSA (susceptibilities pending)" noted on **November 14**. This supports the decision to order the test. This aligns with inclusion criterion 4 regarding suspected infection syndromes showing potential for bacteremia.
**Negative**	Hypothermia	Not appropriate	Appropriate	Appropriate	Progress Notes	The patient meets criteria for severe sepsis as outlined below:<br>1. **Criterion 1 (Severe Sepsis)**: The patient exhibits suspected infection with associated organ dysfunction. The note states: "Creatinine increased to 2.54, which is indicative of renal dysfunction, and the patient is on norepinephrine to support blood pressure."<br>- **Date of the event**: March 28<br>- **Supporting quote**: "...Cr bumped (1.91 > 2.54) after 4 mg IV Bumex..." indicating organ dysfunction related to the suspected infection.<br><br>2. The patient also meets **Criterion 2** as she has a central venous catheter in place, adding to her risk of infection: "Plan to get PICC line placed tomorrow in anticipation of norepinephrine protocol for HRS."<br>- **Date of the event**: March 12<br>- **Supporting quote**: "Plan to get PICC line placed tomorrow in anticipation of norepinephrine protocol for HRS."<br><br>Given these points, the test order is appropriate for this patient.

**Negative**	Atrial fibrillation with rapid ventricular response. No fevers, no chills. Isolated leukocytosis	Not appropriate	Not appropriate	Not appropriate	Progress Notes	The patient does not meet the required inclusion criteria for the test order while also falling under exclusion criteria. Specifically:<br><br>1. **Inclusion Criteria Review:**<br>- **Criterion 1 (Severe Sepsis):** The patient does not exhibit signs of infection with organ dysfunction and does not meet SIRS criteria. Although the patient has leukocytosis (WBC count of 13.4), there is no evidence of fever or suspected organ dysfunction related to infection.<br>- **Criterion 2 (Systemic Signs of Infection + High Risk of Endovascular Infection):** There are no high-risk factors (e.g., ICD/pacemaker, vascular graft) presented in the note.<br>- **Criterion 3 (Systemic Signs of Infection + Asplenia):** No indication of asplenia is present.<br>- **Criterion 4 (Suspected or Confirmed Infection with Specific Syndromes):** The patient does not have syndromes listed, as she lacks specific infections that would qualify.<br><br>2. **Exclusion Criteria Review:**<br>- **Criterion 5 (Syndromes with Low Risk of Bacteremia):** The note indicates potential chronic issues rather than acute infection or severe sepsis; her leukocytosis may be due to non-infectious causes.<br>- **Criterion 6 (Surveillance Testing):** There is no indication that this test is ordered for routine monitoring.<br>- **Criterion 7 (Fever or Leukocytosis Due to Non-Infectious Causes):** The leukocytosis is most likely from the patient’s underlying CKD and chronic issues rather than infection.<br>- **Criterion 8 (Non-Infectious Syndromes):** The patient has cardiac conditions and other chronic illnesses that might explain her current status without suggesting an active infectious process.<br><br>Given these findings, I conclude that the test order is inappropriate, thus yielding a final classification of **False**.
**Negative**	Isolated hypotension	Not appropriate	Appropriate	Appropriate	Progress Notes	The patient meets multiple inclusion criteria for the test order. Specifically, it meets criterion 1 for severe sepsis. The records indicate the patient had suspected infection and associated organ dysfunction: "this patient presents with hypercapnic respiratory failure requiring intubation..." and shows signs of infection, including leukocytosis. Additionally, criterion 4 is satisfied, as there is suspected or confirmed infection with severe pneumonia: "severe

						pneumonia, requiring ventilation or present in a patient with septic shock/severe sepsis." The relevant date is November 21.
**Negative**	Fever, leukocytosis, hyopoxemia, with concerns for VAP	Appropriate	Appropriate	Not appropriate	Progress Notes	The patient meets criterion 1 for severe sepsis. On April 26 the patient was admitted with severe DKA, which is a suspected infection presenting with associated organ dysfunction (acute kidney injury and respiratory failure leading to intubation). The clinic note states: "Pt admitted to MICU, encephalopathic, profound acidemia with electrolyte derangements, found to be in DKA". This supports the conclusion that the patient is experiencing severe sepsis.
**Negative**	Painless jaundice, negative blood cultures within 72 hours	Not appropriate	Appropriate	Appropriate	Progress Notes	The patient meets inclusion criterion 1 for severe sepsis, as he has a suspected infection associated with organ dysfunction and meets SIRS criteria. Specifically, the note states: 'Patient found to have profound acidosis, azotemia, ?acute/chronic renal failure, and painless jaundice' from the evaluation on July 5. This indicates organ dysfunction (renal failure) and shows severe metabolic derangement, supporting the need for further testing due to the high suspicion of concomitant infection. The noted SIRS indications are clear, justifying the test order as appropriate.
**Negative**	Fever and central line	Appropriate	Not appropriate	Not appropriate	Progress Notes	The patient does not meet the inclusion criteria for ordering the test. Specifically, there is no indication of severe sepsis, systemic infection signs, or the presence of specific syndromes strongly associated with bacteremia in the clinical note. Criteria 5 is also applicable as the patient presents with a low-risk syndrome for bacteremia (community-acquired pneumonia) without meeting the standards for severe sepsis. As noted, "Patient in no acute distress. Objective: Patient received supine in bed, NAD." (October 11) indicates no acute medical concerns that would warrant the test. Thus, the test order is deemed inappropriate.
**Negative**	Urosepsis	Appropriate	Not appropriate	Not appropriate	Progress Notes	The patient does not meet any inclusion criteria sufficient to warrant the test order. In reviewing the clinic note, it lacks documentation of severe sepsis or other strong indicators for infection that would necessitate the test. The criteria checked (1-4) show no evidence of severe sepsis or systemic signs of infection aligning with high-risk factors. Specifically, the absence of qualifying symptoms means the patient falls under the exclusion criteria for low risk of bacteremia associated with infection syndromes."

Abbreviations: BCx, blood cultures; CKD, chronic kidney disease; Cr, creatinine; DKA, diabetic ketoacidosis; HRS, hepatorenal syndrome; ICD, intracardiac defibrillator; IV, intravenous; LLE, left lower extremity; MICU, medical intensive care unit; MRSA, methicillin-resistant *Staphylococcus aureus*; NAD, not in acute distress; NGTD, no growth to date; PICC, peripherally inserted central catheter; SIRS, systemic inflammatory response syndrome; WBC, white blood count.
